# Entropy and Entropic Forces to Model Biological Fluids

**DOI:** 10.3390/e23091166

**Published:** 2021-09-04

**Authors:** Rafael M. Gutierrez, George T. Shubeita, Chandrashekhar U. Murade, Jianfeng Guo

**Affiliations:** 1 Science Division, Physics, New York University Abu Dhabi, Saadiyat Island, Abu Dhabi, United Arab Emirates; george.shubeita@nyu.edu (G.T.S.); chandra.murade@nyu.edu (C.U.M.); dannyguo233@nyu.edu (J.G.); 2 Centro de Investigaciones en Ciencias Básicas y Aplicadas, Universidad Antonio Nariño, Bogotá 111321, Colombia

**Keywords:** entropic forces, biological fluids, crowding, polymer’s configurations

## Abstract

Living cells are complex systems characterized by fluids crowded by hundreds of different elements, including, in particular, a high density of polymers. They are an excellent and challenging laboratory to study exotic emerging physical phenomena, where entropic forces emerge from the organization processes of many-body interactions. The competition between microscopic and entropic forces may generate complex behaviors, such as phase transitions, which living cells may use to accomplish their functions. In the era of big data, where biological information abounds, but general principles and precise understanding of the microscopic interactions is scarce, entropy methods may offer significant information. In this work, we developed a model where a complex thermodynamic equilibrium resulted from the competition between an effective electrostatic short-range interaction and the entropic forces emerging in a fluid crowded by different sized polymers. The target audience for this article are interdisciplinary researchers in complex systems, particularly in thermodynamics and biophysics modeling.

## 1. Introduction

Biological fluids, inside living cells, are crowded by a diversity of polymers in the form of chains, made of monomers ranging from 1.5 to 7 Å in length, such as DNA, which is made of nucleotides. The diversity of polymers in a biological fluid may have different configurations depending on their length, temperature, solvent, and crowding [1,2]. Simple models are useful and powerful, but they may not account for specific details that eventually become dominant effects. By simple models, we are referring to those with a specific microscopic or macroscopic perspective, although, in fact, many of them are very complicated. Several of these models are discussed in references [1,2,3,4] and in their corresponding bibliography. The complexity of this work lies in its consideration of the interactions of both microscopic and macroscopic forces to describe the possible configurations of small polymers as emerging patterns with corresponding emerging qualities. For example, emergent medium- and long-range entropic forces may cause polymer compression, but electrostatic short-range forces cause polymer stretching, generating a resourceful competition of forces with non-obvious behaviors and consequences. The outcome of these different, but competing forces is strongly dependent on small variations of the specific circumstances, such as solvent, the nature and size of the polymers involved, and the crowding they generate with respect to other polymers and to themselves.

The quality of the solvent depends on both the chemical compositions of the polymer and the kind of solvent molecules involved. If a solvent has the precise characteristics to cancel the effects of excluded volume expansion or compression, depending on the point of view, the polymer chain will behave exactly as predicted by the random walk or ideal chain model. At short range, the steric effects are non-bonding interactions that influence the configuration and reactivity of all the ions and molecules in the fluid. Steric effects complement and can be considered a part of the electrostatic short-range intrapolymer and interpolymer interactions between monomers from the same polymer, and monomers from different polymers, dictating the shape and reactivity of the polymers [1,2,3,4].

Such richness and complexity of biological fluids allows for sophisticated dynamics, depending on subtle changes of the internal and environmental parameters, including temperature, in a constant feedback. Therefore, the effects of entropy are ubiquitous in biological fluids. In fact, it is indispensable for certain emergent properties, such as molecular displacement, shape changes, molecular couplings, and, in particular, strong feedbacks with the environment, which they permanently change and are a part of, emerging from the ordering and self–ordering processes that give life to inert matter as an emergent quality from the complexity of many interacting simple and non-living, but very dynamic and sensitive, constituents. The ordering power of entropic forces emerges from the tendency of thermodynamic systems to maximize its entropy, which is understood as a tendency to disorder, but towards more probable states. The complexity of a crowded biological fluid must capture the essentials of diversity into the power of organization patterns and structures with new qualities [2,3,4,5]. In this work, we model the complexity of a biological fluid by describing the polymer configuration changes by means of two competing and complementary forces: (i) an expanding short-range force, F_1_, accounting for electrostatic forces, electronic clouds superpositions, and quantum exclusion principle among others, and (ii) a medium and long–range compressive force, F_2_, accounting for entropic forces like polymer packing, clustering, and osmotic pressure among others. These two resultant, effective forces shape the configuration of the polymer and, therefore, the activity and effects of the molecules, which are also dependent on temperature as a complex thermodynamic system.

The traditional measures of polymer configurations are the hydrodynamic radius *R_h_*, obtained from experimental viscosity measures and quasi–elastic light scattering, the radius of gyration *R_g_*, obtained from experimental measures of small angle X–ray scattering, and the end-to-end distance Re-e, obtained from fluorescence resonance energy transfer (FRET), among other techniques. In certain specific conditions, these three different measures have some theoretical approximations and relations. For good solvents *R_h_* ≈ 5/3*R_g_* and *R_g_* ≈ 1/√6 *R_e-e_*. For example, large non-ionic polymers, such as polyethylene glycol (PEG), which has a molecular weight of 6 kg/M, are constituted by slightly less than one hundred monomers, with an *R_h_* ≈ 24 Å or an *R*_g_ ≈ 40 Å at standard biological fluid conditions [5,6,7,8]. On the other hand, small but charged polymers, polyelectrolytes, made of a few monomers, for example a short single strand of DNA of the nucleotide or base T (thymine), pT, may have an *R_g_* of a few to 100 Å, depending on the crowding, number of monomers, and solvent. Therefore, the size, shape, and compactness of polymer configurations may give very different results for the corresponding measures obtained with different techniques [9], depending on small variations in the conditions. Theoretically, the competition of a negative short-range expanding force F_1_, and a positive large range compressing force F_2_, is well described by a Lennard-Jones-like potential, V_L-J_. For a small pT embedded in a fluid crowded by large PEGs, for example, the minimum of the V_L-J_ indicates the thermodynamic equilibrium of the system when the pT acquires specific values of *R_g_*, *R_h_*, and *R_e-e_* as the forces F_1_ and F_2_ work to mold its size and shape. The crowding, measured by the percentage or density of large polymers, does not have to be very high, as 20% is considered standard for biological fluids with small polyelectrolytes and large non-ionic polymers. Small and charged polymers, such as a small pT, have a characteristic stiffness, represented by F_1_, as it is mainly the repulsion force between the negatively charged monomers that constitutes the charged DNA backbone. In contrast, F_2_ includes crowding, the reduction of available volume that effectively increases the concentration of macromolecules and generates an osmotic pressure upon themselves and over the other constituents of the crowded biological fluid. Therefore, the shape of a pT depends on the feedback of these two forces. The different ranges of these two forces result in subtle balances, wherein when the intensity of one increases, the intensity of the other decreases, and vice versa, as the pT changes its configuration and, consequently, changes the results of the measurements and their quantitative relations.

The general description of the polymer configurations, referred to as thermodynamic equilibrium, is not always entirely correct, because in dilute aqueous solutions, the degradation of biopolymers to monomers is always favored in the thermodynamic sense. However, in a biological fluid there always remains certain kinds of polymers: the smaller the polymer, the greater its stability. For example, single stranded pieces of DNA (ssDNA) are increasingly used as probes with diverse purposes because of their stability and robustness. Polymers embedded in biological fluids can persist for extended periods of time in non-equilibrium states via kinetic trapping by folding and self–assembling. At the most fundamental level, polymer chains are pre-organized for folding by geometrically arrayed, self–complementary molecular interactions and geometric propensities to fold, induced by rotameric and steric restraints on conformation. Therefore, with the thermodynamic equilibrium of a small electrolyte, we assume an equilibrium or, more precisely, a mean state that is maintained by the interplay of many microscopic processes resumed in the force F_1_ because they are all of very short range compared with the long-range entropic forces resumed by the force F_2_. This approach may seem too reductive to analyze polymer configurations, but the complexity argument for small polyelectrolytes, which is the organization and self-organization of microscopic diversity into a simpler emerging pattern of larger dimensions, can be useful to the understanding and description of some aspects of the geometry of small polymer configurations in biological fluids.

This work is divided in four sections: Section 2 presents the theoretical framework, Section 3 presents the model calculations and compares experimental and theoretical results, Section 4 provides a discussion and analysis of the results, and, finally, Section 5 presents some conclusions and perspectives.

## 2. Framework and Methods

A polymer is a complex system with many different possible configurations that may be characterized by their size and shape. In particular, if it is a small charged polymer, it is known as a small polyelectrolyte. The length, the solvent, and the crowding concentrations of biological fluids seem to be indispensable for living functions within and among living cells [1,2,3]. The configurations of a polymer can be measured in different ways. The hydrodynamic radius, *R_h_*, is experimentally measurable using the diffusion coefficient, corresponding to the model of hydrated polymer molecules as solid spheres with radius *R_h_* using the Einstein viscosity relation. The root-mean-squared end-to-end distance of a polymer is denoted by *R_e-e_*; at standard conditions it follows that *R_e-e_* ≈ 3.1*R_h_* [6]. For a freely jointed chain, when the scaling factor is *ν* = 1/2, we can write *R_e-e_ = 6*^1/2^*R_g_ = A*_0_ (6*N*)^1/2^, where *R_g_* is the gyration radius, *N* is the number of monomers, and *A*_0_ can be interpreted as the effective length of one monomer. For an ideal chain, *R_e-e_* = *N*^1/2^*l*, where *l* is the Kuhn or persistent length [10]. In general, the scaling relation *R_e-e_* ~ *N^ν^* is considered, where the scaling factor *ν* = 3/5 = 0.6 corresponds to a good solvent, *ν* = 0.33 for a spherical configuration, *ν* = 0.5 for a theta solvent, and ν = 1 for a straight, rope-like polymer. A good solvent generates a pair-wise repulsion for full chain swelling, whereas a regular solvent screens the repulsion between monomers and the chain collapses closer to a spherical configuration. For example, a salt concentration in water as the solvent for polyelectrolytes, such as pT, acts as a poor solvent because it reduces the pair-wise repulsion between the charged monomers of the pT by ionic screening. This solution makes the polyelectrolyte softer and easer to compress into compact configurations by any force that may be present. This is contrary to a good solvent that, in general, is considered to be a substance that increases the stiffness of the polymers to favor the stretched configurations.

The radius of gyration, *R_g_*, is obtained from angular inertia, and it accounts for how the mass of an object is distributed about its center of mass. It can be expressed as *R_g_ = A*_0_*N^ν^*, where *N* is the number of monomers, *A*_0_ is the size or effective length of a monomer, and *v* is the scaling factor that measures the stiffness of the polymer, both of which depend on the salt concentration and were experimentally measured for some polyelectrolytes [7]. *R_h_* is experimentally measurable and, for good solvents, the *R_h_* and *R_g_* may be significantly different. *R_g_* is a model-free measure of the global size of a polymer, and it can be directly determined from small-angle X-ray scattering (SAXS) [8,11,12,13]. In the extreme case where *ν* = 1, the molecular size scales linearly with the number of monomers, suggesting that the monomers in the polymer are rigidly connected, as is the case for pTs and single-stranded DNA (ssDNA) on short length scales. A smaller value of *ν* indicates greater molecular flexibility; in the limiting case where the polymer behaves as a self-avoiding random walk (SAW) chain, it corresponds to *ν* = 0.588 for a large *N* [13]. The steric contribution is included in *A*_0_ and *ν* even if it may or may not depend on salt concentration. Therefore, *R_g_* is a good measure of the size of a small pT, which is experimentally measurable, and it must be a function of: salt (NaCl) concentration *s*, the number of monomers *N*, and the crowding, in mass or volume, percentage of the fluid, *P*. Then, we can write:

(1) $$R_{g}\left(s,N,P\right)=A_{0}\left(s\right)N^{\nu \left(s\right)}F\left(s,N,P\right)\;\;$$

where the first two factors of the right-hand side of the equation have the information of the pT without crowding and *F(s*,*N*,*P*) is the deformation factor, representing the effects of crowding, which also depends on *s*, *N*, and, obviously, *P*. By definition, *F(s*,*N*,0) *≡ F*_0_ = 1 when *P* = 0. As mentioned before, in the absence of long-range interactions, the following mean values relation holds: *R_g_*^2^ = 1/6 *R_e-e_*^2^. The contour length of a polymer, given by *L = NA*_0_, is the polymer stretched by pulling its ends apart. The Kuhn length, or persistent length *l*, defines the stiffness of the polymer and is the length of *N* rigid monomers before the polymer bends. In Table 1, some values of *A*_0_ and *ν* are presented for the corresponding values of salt concentration *s*, as obtained from experimental data [7]. This experimental data was obtained for a few well distributed values of *s*, therefore they can be fitted to the following expression, and we can then obtain extrapolated values of *A*_0_ and ν for continuous values of *s*. From this expression, we can estimate all possible and even idealized theoretical values of *A*_0_ and *ν* for very low and very high concentration values of *s*:

*A*_0_ = 11/4 + *y*/4 and *ν* = 0.794c − 0.0674*y* (2)

where *y* = log10 *s*, 0 *< y* *≤* 3, and 0 *< s* *≤* 20 M is the salt concentration. However, according to the experimental results, *A*_0_ does not have a clear tendency as a function of *s*, but a strong variability for some particular values of *s*. Therefore, for the sake of qualitative estimates throughout this work, *A*_0_(*s*) is normalized to 1 for all values of *s*. This normalization gets rid of noisy effects at the monomer scale and allows us to capture essential features at the polymer scale.

The microscopic forces and their interplay constituting F_1_ may be very complex, including the van der Waals interaction that may change by environmental chemical bonds, temperature increases exclusion volume because of random walk and vibrations, steric effects correspond to very short-range, but non-bonding interactions, like repulsive forces between overlapping electron clouds, attraction by Casimir effect becomes repulsive at certain distances by electron clouds overlapping, among other microscopic complexities of F_1_. However, all of them are strongly depend on microscopic charge distributions and therefore can be reduced or resumed into an electrostatic force strongly dependent on the ion’s concentration provided by the solvent. Then the solvent characteristics, represented by salt concentration, can be directly related with the polymer stiffness as the emergent relevant quality at the relevant scales of the V_L-J_. Thus, through the effective forces F_1_ and F_2_, the model brings up an effective potential from microscopic interactions and entropic forces emergent from the complexity of the system.

Macromolecular crowding is an effect exerted by large molecules on the properties of other large molecules and, indirectly, to themselves and finally to all the other molecules contained in the biological fluid. In summary, F_1_ and F_2_ result in polyelectrolytes, which are less or more flexible, with configurations sometimes difficult to explain in terms of experimental measurements, but they do not collapse to globular forms for large ranges of salt concentration [7]. Electrostatic repulsion tends to disfavor compaction and folding towards a rope-like configuration. Size decreases with increasing salt concentration because the electrostatic repulsion is increasingly screened by salt ions. However, even for high salt concentrations of 1 M, the pT does not collapse into globular forms, indicating that entropic forces are neutralized at some point by other forces like friction, viscosity, and thermal fluctuations as macroscopic expressions of F_1_.

Since the osmotic pressure is an effect of large polymers on polymers of a similar size, the osmotic pressure on small pTs will be more effective with smaller PEGs for the same volume percentage of crowders, as “smaller polymer crowders may be better crowders” [3]. More precisely, polymers are better crowders for similar sized polymers. In addition, the shape of the polymers is important to increase the effective surface of interaction. When the polymers are small, they can no longer be approximated by hard spheres, but instead are more akin to deformable soft spheres with changes of symmetry and dimensionality. These changes can be detected and eventually quantified from the experimental measurements of *R_h_*, *R_g_*, and *R_e-e_*.

The two resulting effective forces F_1_ and F_2_ can be represented by a Lennard-Jones-like potential, *V_L-J_*, where *m* and *n* are characteristic parameters of the specific potential, defining the range relation between the two forces:

(3) $$V_{L-J}\left(r\right)=a\varepsilon \left[{\left(\frac{\sigma }{r}\right)}^{m}-{\left(\frac{\sigma }{r}\right)}^{n}\right]$$

or

(4) $$V_{L-J}\left(r\right)=\varepsilon \left[{\left(\frac{r_{m}}{r}\right)}^{m}-b{\left(\frac{r_{m}}{r}\right)}^{n}\right]$$

**Figure 1:**
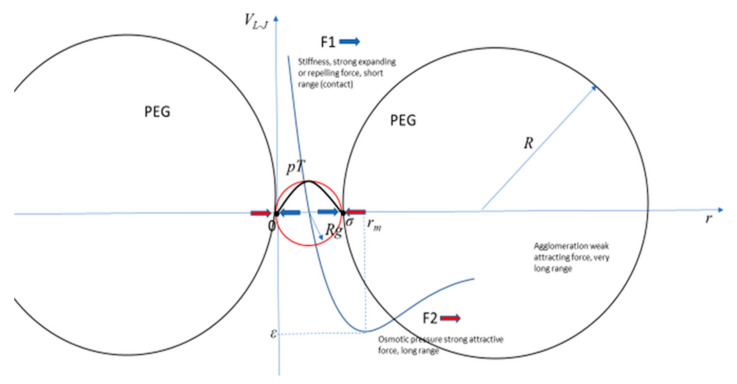
A general Lennard-Jones-like potential representing the forces F_1_ and F_2_ in a system of a small polyelectrolyte pT under the pressure of large non-ionic polymers PEG.

Traditionally, *r* is the distance between two interacting particles, *ε* is the depth of the potential well, and *σ* is the distance at which the particle-particle potential energy is zero, and has its minimum at a distance of *r_m_*, where the potential energy has the value −*ε*. The interpretation in this work for *r* is not the distance between two particles, but the size of the pT, which changes under the pressure of the PEG, with a resistance to changes depending on the stiffness of the pT, measured by the scaling parameter, that is dependent on the salt concentration, *ν*(*s*). If the reference point is one extreme of the *pT*, and *r* is the distance to the other extreme, then *r_m_* = (*m/n*)^1/*m-n*^*σ* represents the compression or expansion of the pT under the two competing forces. The condition *m > n* represents the short- and long-range forces, respectively. The other two parameters, *a* and *b*, depend on the values of *m* and *n*. For the standard Lennard-Jones potential, *m =* 12, *n* = 6, *a* = 4, and *b* = 1. What justifies the application of the Lennard-Jones potential to study this problem is the large difference between the ranges of the two forces F_1_ and F_2_ as they were defined, respectively, as negative, or repulsive-expanding, and positive, or attractive-compressing, of the small polyelectrolyte. The system with large polymers, PEG, and a small polyelectrolyte, *pT*, is depicted in Figure 1 with the corresponding *V_L-J_.* Under this condition *m* and *n* can have many different values compared to the most common and original Lennard-Jones potential’s values of *m* = 12 and *n* = 6. The forces F_1_ and F_2_ are given by the variation of the potential, where a negative sign indicates repulsive force and a positive indicates attractive force, or stretching and compressing the pT, respectively:

(5) $$\frac{-dV_{L_{J}}\left(r\right)}{dr}=-F_{1}\left(r\right)+F_{2}\left(r\right)$$

From Equations (3) and (4), the equilibrium of the system is different depending on *s*, *N*, and *P*, which define the relations between the two forces, determined by:

(6) $$r_{m}={\left(\frac{m}{n}\right)}^{1/\left(m-n\right)}\sigma$$

Different values of the relative intensities and ranges of the two forces, represented by the powers *m* and *n*, correspond to specific values of *a* and *b* and a quantitative relation between *r_m_* and σ. The factor *F_mn_*:

(7) $$F_{mn}={\left(\frac{m}{n}\right)}^{1/\left(m-n\right)}$$

measures the contraction of the pT, proportional to its length when there is not crowding. When *F_mn_ >* 1, the pT is compressed and when *F_mn_* < 1, the pT is stretched, and in both cases the configuration of the polyelectrolyte is changed at the same time that it changes the effects of the two forces upon itself.

From the diagram of the V_L-J_ and the corresponding definitions, we identify *R_e-e_* = σ and *r_m_ = βσ = βR_e-e_*, where *β* is the measure of change of size of the pT under the osmotic pressure from crowding; *R_e-e_ = σ* is the size of the compressed pT and *r_m_ = βσ = βR_e-e_* is the measure of the pT without crowding. This approximation is theoretically valid for any configuration of the pT, considering that *R_e-e_* measures the characteristic size of the pT and its extremes tend to the surface of the volume, defined by the polymer, as the statistically more probable locations for them. Therefore, the compression factor depends on the number of monomers constituting the pT, *N*, the salt concentration *s*, and the crowding percentage, *P*, given by:

(8) $$\beta \left(s,N,P\right)={\left(\frac{m}{n}\right)}^{\frac{1}{m-n}}$$

From Equations (3) and (4), with their derivatives, equal to zero, as the equilibrium condition, and Equations (5)–(7), we get *a = βm* and *b = βm-n*. For example, for a pT approximated by an ellipsoid with reduced symmetry instead of a sphere (the ellipsoid with the highest symmetry), from Equations (3) and (4), the forces F_1_ and F_2_ must have the approximate relation given by:

(9) $$F_{1}=F_{2}\left(1-\frac{2d}{\sqrt{d^{2}+c^{2}}}\right)$$

where *d* is the largest axes and *c* is the other two equal and smaller axes of the ellipsoid. In the case of a sphere, when *c = d*, we obtain F_1_ = −0.41F_2_, and for an ellipsoid with *c = d*/2 we obtain F_1_ = −0.78F_2_. These simple calculations show the changes that the feedback between the pT geometry and the forces can produce on the pT’s configuration. For a larger F_1_ relative to F_2_, the configuration of the pT tends to lose symmetry, changing from a sphere to an ellipsoid.

In general, since the entropic forces are isotropic, they act as a compression or compacting force upon the pT, but the monomers of the extremes of the pT have more degrees of freedom than the other monomers, and tend to move away from the others towards the surface of the volume occupied by the polymer as a more probable configuration. In contrast, F_1_ is directional and tends to stretch the polymer towards a more elongated, less spherical, and less symmetric configuration that also generates a positive feedback with F_2_ to make the pT configuration still less symmetric and flatter, tending to reduce dimensionality from 3D to 2D, towards a parabola- or horseshoe-like configuration and, finally, to the extreme 1D configuration of a stretched rope. For smaller pTs, this tendency to dimensionality reduction, generated by the feedback of the forces and the geometry of the pT, is stronger.

The idealized configurations corresponding to different stages of symmetry and dimensionality loss may be reduced to the following:

1. Rope-like shape: is the maximum extended idealized configuration with all the monomers in line with the extremes maximally separated from each other. It is the dominant tendency of a strongly charged polyelectrolyte with strong repulsion between monomers, and is a very stiff polymer that is difficult to bend. This configuration is almost 1D. Its total length will be approximately equal to Re-e(𝑠,p) = 𝐴_0_(𝑠)N.
2. Parabola- or horseshoe-like shape: is a less extended configuration, but still dominated by F1 repulsion among monomers, weakened by the larger distance between the extreme monomers. The polymer is still stiff, but the entropic packing force becomes relatively more important and the extreme monomers can get closer, thereby bending the polymer. Compared with the rope-like shape, the dimensionality increases to an almost 2D configuration. Its total length is still approximately equal to 𝐴_0_(𝑠)N, but R_e-e_(𝑠,N) has a more complex dependency on s and N.
3. Ellipsoid shape: when the polymer is soft and longer, the entropic packing force makes the monomers fill the space between the two extremes more homogenously, thereby developing an elongated volume. The extremes still tend to be at the end of the longer axes of the elongated shape because it is where they have fewer constraints and a maximum probability to be located there. Its dimensionality increases to 3D.
4. Spheroid shape: when the polymer is softer and longer, the entropic packing force makes the monomers homogeneously fill the space between the extremes, but with maximum symmetry because the preferred directions, defined by the interactions between monomers, lose importance, while the extremes stay close to the surface for the same entropic reasons mentioned previously. The spherical symmetry of the volume occupied by the monomers becomes definitively 3D in nature. The monomers can be more or less dispersed in the spheroid volume, which is occupied by polymers that tend toward a compact and solid sphere shape. When the compression force, F_2_, overcomes the expansion force, F_1_, the volume occupied by the monomers becomes increasingly symmetric and asymptotically tends toward the most compact and further incompressible configuration, while limited by temperature and steric interactions between monomers. Larger polymers always have more room for compression. For a compact sphere, R_e-e_ ~ 2R_g_.

The radius of gyration, *R_g_*, is defined as the mean square distance of the monomers to the center of mass of the polymer:

(10) $$R_{g}={\left(\frac{1}{N}\sum_{i=1}^{N}r_{i}^{2}\right)}^{\frac{1}{2}}$$

where *N* is the number of monomers and *r_i_* is the distance from the *i*-th monomer to the polymer’s center of mass, considering all the monomers with mass equal to one. As indicated previously, the radius of gyration, *R_g_*, was measured for different values of s and *N* with *p* = 0, following the scaling relation [6]:

(11) $$R_{g}\left(s,\;N,0\right)=A_{0}\left(s\right)N^{\nu \left(s\right)}$$

By defining *α(s*,*p*) as *R_g_ = α R_e-e_*, the proportionality factor between these two measurements of a polymer, theoretical and experimental limits can be better compared to understand the configuration changes of a pT under variations of *s*, *N*, and *P*, causing changes of F_1_ and F_2_. For high values of *s*, the pTs can be considered freely jointed polymers, i.e., stochastic chains in 3D with *α* ≈ 1/√6 ≈ 0.408, following a random walk in 3D in the absence of any force: F_1_ = F_2_ = 0. Different values of *α* indicate how different the corresponding polymer configuration is compared to that of the freely jointed polymer.

When the monomers are considered hard spheres with no electrical charge of any kind, F_1_ = 0 and there is no crowding, but with interpolymer entropic packing force, which means F_2_ is weak, the polymer tends to a symmetric spheroid 3D volume randomly occupied as a tight random walk of the monomers, with the extremes tending to the surface by the entropic reason explained above. Depending on the temperature T, the spheroid may be more or less compact, tending toward a dense and solid sphere for a very low T. In such conditions, the polymer can be well approximated by a solid sphere of radius *R* with *R_g_ = (*6/5)^1/2^*R ≈* 1.1*R*, corresponding to the mathematical ideal radius of gyration of a solid sphere with radius *R* obtained from the corresponding 3D gyration tensor. If we estimate *R* by making the volume of *N* small solid spheres of unit radius, equal to the volume of a solid sphere of radius *R*, we obtain *R* *≈* *∛N*. Then, for a polymer made of *N* monomers with a compact sphere configuration, we obtain *R_g_ ≈ (*6/5)^1/2^*N*^1/3^ *≈* 1.1*N*^1/3^ indicating that the radius of gyration is 10% larger than the radius of the sphere. Considering the entropic tendency of the extremes of the polymer to be in diametral opposite sides of the sphere, the end-to-end distance would be given by:

(12) $$R_{e-e}\approx 2R=2N^{\frac{1}{3}}=2\sqrt{\frac{5}{6}}R_{g}=1.8R_{g}$$

for a solid sphere polymer configuration of *N* monomers. For ring-like and disc-like tight and idealized configurations of *N* monomers, *R_g_ ≈* √2*N* and *R_g_ ≈ N*, respectively.

A second theoretical expression for *R_g_*, resulting from the “wormlike” chain model [14], is given by:

(13) Rg=lLp3−Lp2+2Lp3l−2Lp4l21−e−lLp1/2

where *L_p_* is the persistent length, *l = N* is the contour length with *N* as the number of monomers and the effective monomer length equal to one. For the maximum stiffness, *L_p_ ~ N*, and a very large polymer, *N→∞*, we obtain *R_g_* *≈ N*/4, which is the same result for the minimum and maximum stiffness of very small polymers, *N→*1, because *L_p_→N→*1. This means that small polymers are simple and do not have much room for stiffens variability, as it is always close to the maximum, *L_p_* *≈* 1. The minimum stiffness, *L_p_ =* 1, for very large polymers, *N→∞*, gives *R_g_→(N*/3)^1/2^.

## 3. Calculations

### 3.1. Small pT Configurations

In the context of this work, the configurations of the small polyelectrolyte, pT, depend only on the parameters *s*, *N*, and *P*. As indicated, we expect a reduction of *R_g_* for increasing *s* and independently of *P*, both of which depend on *N* in a different way. The idealized configurations are related, with the scaling exponent of *ν*(*s*) = 3/5 = 0.6 for a good solvent, *ν*(*s*) = ½ = 0.5 for a theta solvent, and *ν*(*s*) = 1/3 ≈ 0.33 for a poor solvent with the corresponding values of the solvent concentration *s*. Therefore, the approximation of a small polyelectrolyte to a spheroid is much better when the solvent is rather poor and the number of monomers is not too small.

**Figure 2:**
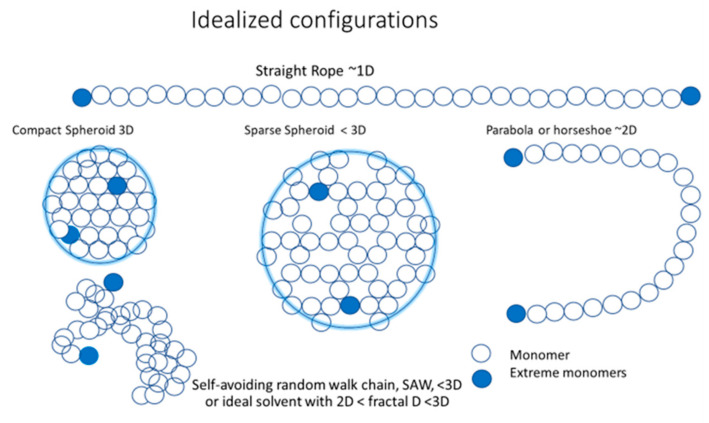
Five idealized configurations of small electrolytes: 3D compact or dense spheroid (DS); less than 3D sparse spheroid (SS); less than 2D parabola (Pa); 1D straight rope (SR); and the self–avoiding random walk (SAW), which, in an ideal solvent, can become a fractal object with reduced symmetry, reduced dimensionality, and low density, but maximizing the surface contact with the polymer solvent.

Figure 2 presents an idealization of five configurations: a straight rope-like configuration (SR) that is almost one-dimensional, ~1D; a parabola- or horseshoe-like configuration (Pa) that is almost two-dimensional, ~2D; a sparse spheroid (SS) with dimensionality less than three, <3D, a compact or dense spheroid (DS) that is three-dimensional, 3D; and, finally, the more realistic self–avoiding random walk (SAW) configuration, corresponding to a very good solvent with a dimension < 3D. The SAW configuration is the real optimal configuration for an ideal solvent, providing maximal surface contact with it, and it may have a fractal dimension f where 2D < fD < 3D.

With Equations (9)–(11) we can estimate, in three different ways, the radius of the gyration, *R_g_*, and use approximations of the corresponding values of the end-to-end distance *R_e-e_* in order to obtain new relevant information to understand the real configurations of the small electrolytes at different conditions of *s*, *N*, and *P.*

### 3.2. Radius of Gyration for Small pT Configurations

**Figure 3:**
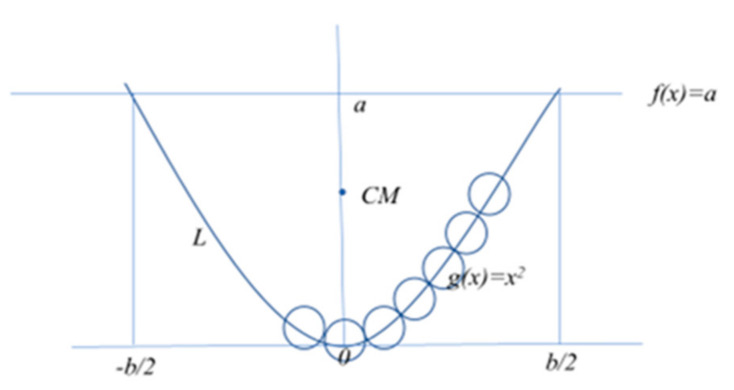
Building a polymer parabola-like configuration, *y(x) = x*^2^, monomer by monomer.

Parabola configuration (Pa): for a polymer constituted by *N* unit length monomers with a 2D symmetric parabola configuration, *y*(*x*) = *x*^2^, we first find the center of mass given by *x*_0_ = 0 and *y*_0_ = (50/320)*b*^2^ = 0.18*b*^2^, where *b* is the end-to-end, *R_e-e_*, distance truncated at *y = a*. From the formula to calculate the length, *L*, of such a segment of the parabola, which must be *L = N*, we obtain the increment in the *x* and *y* directions, *∆x* and *∆y*, respectively, when a new monomer is added to the parabola configuration. Then, the distance of the *N* polymer to the center of mass (*x*_0_, *y*_0_) is given by *r_i_* = ((*x*_0_*−x_i_*)^2^ + (*y*_0_−*y_i_*)^2^)^1/2^, where *x_i_ = x_i−1_* + *∆x* and *y_i_* = *y*_*i*−1_ + *∆y*, with *i* = 1,*…*, *N*/2 for an even *N* or *i* = 0, *…*, *N−*1/2 with *r*_0_ = 0 if *N* is odd. For such parabola configurations as depicted in Figure 3, Equation (9) takes the specific form of:

(14) $$R_{g}={\left(\frac{1}{N}\left[\sum_{i=1}^{p/2}{\left(2r_{i}\right)}^{2}\right]\right)}^{\frac{1}{2}}$$

The estimate of the *r_i_* depends on the characteristics of the parabola and can be geometrically elaborated. The factor 2 of the *r_i_* appears because of the 2D symmetry of the parabola configuration *y*(*x*) = *x*^2^, where the unfold length of the polymer is *N* for *N* monomers of unitary length and mass.

**Figure 4:**
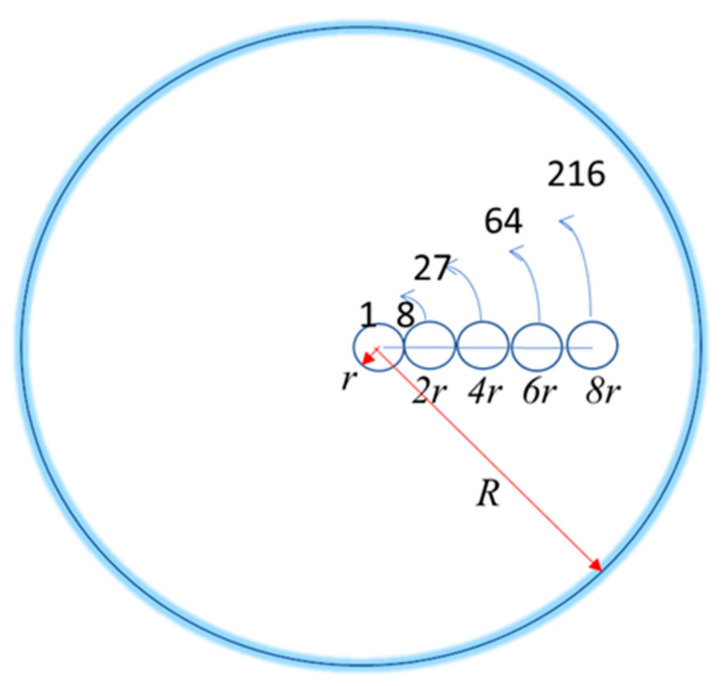
The maximum number of monomers of radius *r* that fit inside a DS polymer configuration of radius *R = r*, 2*r*, 4*r*, 6*r*, 8*r*.

Dense sphere configuration (DS): in this case, Equation (9) for *R_g_* can be written as:

(15) $$R_{g}={\left(\frac{1}{N}\left[n_{1}r_{1}^{2}+n_{1}r_{2}{}^{2}+n_{1}r_{3}{}^{2}+n_{1}r_{4}{}^{2}+\ldots +n_{1}r_{l}{}^{2}\right]\right)}^{\frac{1}{2}}$$

where *r_i_ =* 2(*i*−1*r*) for *i =* 1, 2, *N* is the distance of the *i*–th monomer’s center to the polymer’s center of mass with a sphere configuration, and *n_i_* is the maximum number of monomers that can fit at such a distance from the center in order to have the maximum spherical symmetry. It is obtained by dividing the spherical volume occupied by the polymer *V_R_ =* 4/3*πR*^3^ by the spherical volume occupied by one monomer *V_r_* = 4/3*πr*^3^. With *r =* 1, the corresponding values for a spherical symmetric configuration are *r*_1_ = 0 and *n*_1_ = 1, *r*_2_ = 2 and *n*_2_ = 7, *r*_3_ = 4 and *n*_3_ = 19, *r*_4_ = 6 and *n*_4_ = 37, up to the sum of all *n_i_* equal to *N*, the total number of monomers. Figure 4 shows the maximum number of monomers by layer of the DS configuration. Then, the maximum number of monomers of radius *r* that can fit in the polymer volume of radius *R* is *V_R_/V_r_ = R*^3^*/r*^3^, where *R* increases by units of 2*r*. For *r =* 1, the maximum number of monomers of each layer are respectively 1, 8 − 1 = 7, 27 − 8 − 1 = 18, 64 − 27 − 8 − 1 = 28, 216 − 64 − 27 − 8 − 1 = 116, 1000 − 116 − 28 − 18 − 7 − 1 = 830, and so on.

Thus, for the DS we can rewrite Equation (9), and Equation (14), with all *r_i_ = r =* 1, reduces to:

(16) $$R_{g}={\left(\frac{1}{N}\left[0+7{\left(2r\right)}^{2}+19{\left(4r\right)}^{2}+37{\left(6r\right)}^{2}+152{\left(8r\right)}^{2}+\ldots +n_{l}r_{l}{}^{2}\right]\right)}^{\frac{1}{2}}$$

Sparse sphere configuration (SS): corresponds to Equation (15), but the *n_i_* are smaller than those for DS and the maximum *r_i_* may be larger to fit the same number of monomers tightly packed in a DS configuration. The *R_g_* values for a DS may vary from those very close to the *R_g_* of a DS, to those corresponding to a SAW embedded in a spherical volume with a very low density of monomers. In a ring configuration (Ring) of radius *R*, all the monomer distances to the center of mass are the same, *r_i_ = R* for all *i =* 1, *N* and *R* is estimated dividing the perimeter of the ring, 2*πR*, by the diameter of one monomer, 2*r*. Applying Equation (9) with *N* = 19 and all the *r_i_ =* 6*r* and all the *n_i_ =* 1 for *i =* 1 to 19, we obtain *R_g_* = 6*r*, which is much larger than the corresponding values for a compact sphere.

Straight rope (SR) and SAW: the application of Equation (9) to SR and SAW are straightforward, in the second case considering the *r_i_* with a random angle and the constraint defined by the projection 1D to be *R_e-e_*. In the following tables we present the different results for *R_g_* estimated from the three Equations (9), (10) and (12).

### 3.3. Measures of Small pT Configurations

Table 2 presents the results of *R_g_* using Equation (10) for polymers of *N* monomers, with stiffness *ν*(*s*), for the different values of *s* from Table 1, along with the corresponding values of *R_h_* and *R_e-e_* as estimated by the approximation of large polymers, but depending on salt concentration *s*. In the indicated conditions, the total unfolded length of the polymer is *N*, and for the DS approximation for the radius is given by *R = N*^1/3^.

Table 3 presents the results of the radius of gyration *R_g_*, estimated for SAW with the “wormlike chain” model, Equation (12), for different persistent lengths, *L_p_*, representing the stiffness of the polyelectrolyte: a larger *L_p_* corresponds to a stiffer polymer, and the effective monomer length is normalized to unity. For large polymers, *N→∞*, the minimum stiffness, *L_p_* = l, gives *R_g_ ~ (N*/3)^1/2^, and the maximum stiffness, *L_p_→N*, gives *R_g_ ~ N*/4. For small polymers, *N*→1, the maximum and minimum stiffness are about the same as *R_g_ ~ N*/4.

Table 4 presents the results of *R_g_* for dense (DS) and sparse spheres (SS), parabola (Pa), and straight rope-like (SR) ideal small polymers for the same values of *N*, estimated as the mean square distance of the monomers to the center of mass, Equation (9). The SS is a sphere with a radius *R + r*, where *R* is the radius of the corresponding DS and *r* is the radius of one monomer.

## 4. Results and Discussion

The previous calculations allowed us to compare the changes of the different measurements depending on the parameters *s*, *N*, and *P* and from the point of view of each measurement *R_h_*, *R_e-e_*, and *R_g_* as estimated with different methods. This comparison revealed changes in the size, geometry, and dimensionality of the small pT configurations, which is new and important information necessary to understand the behavior of small polyelectrolytes in biological fluids and the emerging qualities they may contribute to the living cell. However, further studies are necessary in order to have a better quantitative assessment of the corresponding measures and precise quantitative data of the size, geometry, and dimensionality of the small polymers as constituents of biological fluids. Table 5 presents a synthesis of the different measurements of *R_g_* and the corresponding approximations of *R_h_* and *R_e-e_.* For the experimental measurements of *R_g_*, two idealistic extremes were considered: one with a very small salt concentration, *s =* 1 mM, and another with a very large salt concentration, *s =* 10 M. These two limits were intended to compare with the idealist configurations DS, SS, Pa, and SR, simulating the stiffness of the polymer by the ideal qualities of the solvent that may change the output of the forces F_1_ and F_2_. For example, for a small polymer DS configuration, *N~*20, the persistent length must be small, *L_p_ <* 3, and the equivalent salt concentration must be very high, *s >* 10 M. This indicates a compromise between the highest stiffness that a small polymer can have and the highest stiffness that a solvent can provide. However, as a nonlinear feedback process, the interplay between the different ranges and intensities of F_1_ and F_2_ can substantially change the V_L-J_ and, therefore, its critical points will have more alternatives with higher probabilities for larger polymers. However, when the size of the polymer increases and the solvent capacity to soften the polymer is reduced, the results show a tendency of mixed configurations with less symmetry, less spatial density and less homogeneity of the distribution of monomers in space. Thus, the polymer’s stable configurations become more diverse and complex, reducing the probability of the more symmetric and compact states, in particular when the temperature increases, giving energy and instability oscillations to the monomers. For example, for larger polymers, *N* ~ 60, and very small concentrations of solvent, the configurations tend to correspond more to very sparse SS with a tendency to SAW, highly reducing the symmetry and dimensionality of the configuration and corresponding to larger persistent lengths, *L_p_ > N*/5. These changes of configuration correspond to changes of the forces F_1_ and F_2_ and their relations, which in its turn generate further changes in the configurations until a thermodynamic equilibrium is achieved. The shape and size changes of the polyelectrolyte configurations eventually may take the forces F_1_ and F_2_ from competition to cooperation, increasing *R_e-e_* when it is not expected.

Ideally, when *s* is very large and tends towards saturation, the stiffness of the polymer tends to be negligible, thereby reducing *ν* towards the lower limit, with ν(s) > 10 M ≈ 1/2 = 0.5 corresponding to an ideal solvent. The configuration of such a polymer strongly depends on any small interacting force and is unstable at high temperatures. It has a strictly *L_p_ =* 1 only limited by the hard sphere exclusion of neighboring monomers.

When short range steric, electrostatic, electron clouds exclusion, and any other repulsive forces are considered, *L_p_* increases and not all the nearest neighboring locations of a monomer are equally probable, F_1_ is a short-range repulsion between monomers, including the two extreme monomers being pushed towards the surface of the volume occupied by the polymer, increasing *R_e-e_*. Therefore, the stiffness of the polymer is increased, *ν*(*s*) ≈ 1 M ≈ 3/5 = 0.6, corresponding to an ideal chain or self–avoiding random walk chain (SAW), which is very soft for long and medium lengths, but stiff for short lengths, increasing the persistent length to *L_p_ >* 1; at such lengths the repulsion of F_1_ dominates the contraction of F_2_. If this local stiffness is very strong and could propagate to the whole polymer, it would stretch to its maximum extent like a rigid, straight rope with *L_p_ = N*, with *N* the number of monomers of unitary length. With this configuration, the polymer loses all its remaining 3D isotropic symmetry and becomes a 1D straight rope of aligned monomers. In the absence of any other force or thermodynamic perturbation, it can be represented by a total absence of solvent and, therefore, the maximum stiffness with *ν (s ≈* 0) *≈* 1. In this idealized situation, the monomers strongly repel each other stretching the polymer to its maximum length and *Lp = R_e-e_ = N*, the contour length of the polymer.

When the polymer is very soft with an ideal solvent and the short range steric, electrostatic, electron clouds exclusion, and any other repulsive force are dismissed, F_1_ ~ 0, the complexity of the system allows for the emergence and dominance of the packing or agglomeration force as a medium- and long-range entropic force relative to the size of a monomer. This tendency, formally represented by a force, emerges from the thermodynamic potential generated by the tendency to increase entropy towards configurations with larger probability, namely those with more equivalent spatial monomer distributions, which correspond to the more compacted globular configurations. These configurations are the most symmetric and isotropic; they are fully 3D spatial distributions of monomers occupying a spheroid volume in space. The packing force as one constituent of F_2_ is relatively weak and long range but persistent, making the globular configurations inevitable when the other conditions allow it. In these configurations, the only constraint of the monomers is the directional chemical bonds between neighbor monomers to conform the polymer. By symmetry, isotropy and entropy, the tight globular configurations must have a spheroid shape, with the extreme monomers tending to the surface of the volume, with an approximated radius *R = N*^1/3^, obtained from *V_p_/V_m_*, where *V_p_ =* 4/3*πR*^3^ is the volume of the polymer and *V_m_ =* 4/3*πr*^3^ is the volume of a monomer, with radii *R* and *r* respectively, considering *r =* 1. In 3D, the spheroid compacity generated by the entropic packing force reduces the persistent length because the angle between nearest neighbors always changes, making *L_p_ <* 1. This can be seen or represented as a further decrease in the stiffness of the polymer with a smaller *ν*, emulating a much larger salt concentration *s*, *ν(s→∞)* ≈ 1/3 = 0.33.

The dense spheroid (DS) has the maximum symmetry and is fully 3D, while the sparse spheroid (SS) can have different degrees of sparsity, reducing symmetry and dimensionality from 3D up to configurations contained in a 2D disc or even in a ring or incomplete ring with a parabola (Pa) or horseshoe-like configuration, and dimensions even lower than 2D towards a straight rope (SR) with a minimum symmetry and dimensionality of 1D. The self–avoiding random walk (SAW) is a more realistic configuration, but also more complex and with diverse equivalent and non-equivalent configurations. Therefore, it is a highly probable configuration, but difficult to distinguish in its specific differentiated, but similar forms because they may have very similar values for all possible measurements. These configurations have a fractal dimension (fD) where f may be < 2 or < 3, significantly increasing and maximizing, even more so than the straight rope, the surface contact of the polymer with the solvent. Along with this local configuration of the monomers with their own local stiffness, a global, larger scale shape is organized, such as a parabola or horseshoe configuration, which may be much more sensitive to F_2_ and, in particular, in the direction of changing *R_e-e_* and its relations with *R_g_* and *R_h_*. This geometrical effect changes the value of the compression factor *F*(*s*,*N*,*P*) in Equation (1) or, equivalently, the factor *β* relating *R_g_* and *R_e-e_*. This is all a consequence of a nonlinear feedback process between the forces F_1_ and F_2_ with the geometry of the polymer configuration that the forces themselves build.

## 5. Conclusions and Perspectives

This work presents a qualitative interpretation of the diversity of small polyelectrolyte configurations using a simple model based on a Lennard-Jones-like potential alongside diverse experimental and theoretical measures of small polymers in a biological fluid. Despite, or perhaps as a result of, its simplicity, which is a consequence of a diverse complexity of forces resumed into two effective forces, the model provides new useful information about the probable configurations of small polymers in a biological fluid. This information was obtained from the competition and cooperation of the effective short-range microscopic force and the long-range entropic force, which emerges from the complexity and richness of the biological fluid. The competition and, in some circumstances, cooperation of the two forces, generates a rich diversity of small polymer configurations that may give the living cell resources to perform its functions by organizing and allowing self-organization patterns, structures, and dynamics with unexpected results, such as the sudden changes to the effective stiffness of the small polymers. Further efforts will provide the more quantitative and precise results necessary to determine the specific values of the parameters and variables that generate new emergent qualities of the biological fluid, and the corresponding verification, and even prediction, of experimental measurements in the complexity of biological fluids.

## Figures and Tables

**Table 1:** Experimental data and extrapolated values of *A*_0_ and *ν*, using Equation (2), for salt concentrations 0 < *s ≤* 20 M.

Scheme 1	1 mM	12.5	25	125	225	525	1 M	10 M	20 M
*A*_0_(*s*)	2.750	3.024	3.100	3.274	3.38	3.430	3.500	3.750	4.900
*ν*(*s*)	0.794	0.720	0.700	0.653	0.636	0.607	0.592	0.524	0.504

**Table 2:** Approximated values of *R_g_*, *R_h_*, *R_e-e_*, and *R* of small polyelectrolytes of *N* = 20, 30, 40, 50, and 60 monomers for different salt concentrations of *s* = 12.5, 25, 125, 225, and 525 mM. With normalization respect to *A*_0_(*s*), the radius of gyration is *R_g_ = N^ν(s)^* with the corresponding values of *ν*(*s*) given in Table 1, a hydrodynamics radius *R_h_* = (5/3*R_g_*)^1/2^, an end-to-end distance of *R_e-e_* = 3.1*R_h_*, the total unfolded length, *L = N*, and the radius of a compact sphere of *N* monomers of radius *r* = 1 given by *R = N*^1/3^.

*N*	20		30	40	50	60
Measure	[*s*] = *m* M, *ν*(*s*)					
*R_g_ = N^ν*(*s*)*^*	12.5, 0.720	8.8	11.7	14.5	17.0	19.4
	25, 0.700	7.9	10.5	12.8	14.9	16.9
	125, 0.653	7.1	9.2	11.1	12.8	14.4
	225, 0.636	6.5	8.4	10.0	11.5	12.9
	525, 0.607	6.2	8.0	9.5	11.0	12.0
*R_h_* = (5/3*R_g_*)^1/2^	12.5, 0.720	3.8	4.4	4.9	5.3	5.7
	25, 0.700	3.6	4.2	4.6	5.0	5.3
	125, 0.653	3.4	3.9	4.3	4.6	4.9
	225, 0.636	3.3	3.7	4.1	4.4	4.6
	525, 0.607	3.2	3.6	4.0	4.3	4.5
*R_e-e_* = 3.1*R_h_*	12.5, 0.720	11.8	13.7	15.2	16.5	16.6
	25, 0.700	11.3	13.0	14.3	15.5	16.5
	125, 0.653	10.6	12.1	13.3	14.3	15.2
	225, 0.636	10.2	11.6	12.7	13.6	14.4
	525, 0.607	10.0	11.3	12.3	13.2	14.0
*R* = *N*^1/3^		2.7	3.1	3.4	3.7	3.9

**Table 3:** The radius of gyration, *R_g_*, estimated with the “wormlike chain model”, Equation (13), for different persistent lengths, *L_p_*, representing the stiffness of the polyelectrolyte: larger *L_p_* corresponds to a stiffer polymer. The effective monomer length is normalized to unity. For large polymers, *N*→∞, the minimum stiffness, *L_p_ = l*, gives *R_g_ ~ (N*/3)^1/2^, and the maximum stiffness, *L_p_→N*, gives *R_g_ ~ N*/4. For small polymers, *N→*1, the maximum and minimum stiffness are about the same and *R_g_ ~ N*/4.

*R_g_*									
*Lp*	1 min. Stiffness	2	3	4	5	(*N*/10)	(*N*/5)	(*N*/2)	*N* max. Stiffness
*N*									
20	2.4	3.2	3.6	4.0	4.2	(2) 3.2	(4) 4.0	(10) 5.0	5.3
30	3.0	4.1	4.8	5.3	5.7	(3) 4.8	(6) 6.0	(15) 7.3	8.0
40	3.5	4.8	5.7	6.3	6.9	(4) 6.3	(8) 7.9	(20) 9.7	10.5
50	4.0	5.4	6.5	7.3	7.9	(5) 7.9	(10) 9.9	(25) 12.0	13.1
60	4.4	6.0	7.2	8.1	8.9	(6) 9.5	(12) 11.9	(30) 14.5	13.8

**Table 4:** *R_g_* for dense (DS) and sparse spheres (SS), parabola (Pa), and straight rope-like (SR), ideal small polymers of *N* = 20, 30, 40, 50, and 60 monomers, estimated as the mean square distance of the monomers to the center of mass, Equation (9). The SS is a sphere with radius of *R + r*, where *R* is the radius of the corresponding DS and *r* is the radius of one monomer.

*N*		*R_g_*				
	Dense Sphere (DS) of approx. R = *N*^1/3^	*R* = *N*^1/3^	Sparse Sphere (SS)	Parabola (Pa)	Straight Rope (SR)	Ring *Rg* = *R* = *N*/2π
20	3.3	2.71	4.7	3.5	11.53	3.2
30	3.8	3.11	5.2	4.5	17.31	4.8
40	4.5	3.42	6.3	5.4	23.08	6.4
50	4.9	3.68	6.6	6.7	28.86	8.0
60	5.3	3.91	9.6	7.0	34.63	9.5

**Table 5:** Comparison of *R_g_* values estimated from experimental data and theoretical models with the corresponding *R_h_* and *R_e-e_* approximations for small pTs constituted by *N* = 20, 30, 40, 50, and 60 monomers of unit length and mass and different salt concentrations *s*.

*N*	20		30	40	50	60
Measure	[*s*] = *m* M, *ν*(*s*)					
*R_g_* = *N^ν(s^*^)^ Equation (10)	1, 0.794	10.8	14.9	18.7	22.3	25.8
	12.5, 0.720	8.8	11.7	14.5	17.0	19.4
	25, 0.700	7.9	10.5	12.8	14.9	16.9
	125, 0.653	7.1	9.2	11.1	12.8	14.4
	225, 0.636	6.5	8.4	10.0	11.5	12.9
	525, 0.607	6.2	8.0	9.5	11.0	12.0
	1000, 0.592	5.9	7.5	8.9	10.1	11.3
	10,000, 0.524	4.8	5.9	6.9	7.8	8.5
*R_g_* Equation (9)	DS	3.3	3.8	4.5	4.9	5.3
	SS	4.7	5.2	6.3	6.6	9.6
	Pa	3.5	4.5	5.4	6.7	7.0
	SR	11.5	17.3	23.1	28.9	34.6
*R_g_* Equation (13)	*L_p_* = 1	2.4	3.0	3.5	4.0	4.4
	*L_p_* = 2	3.2	4.1	4.8	5.4	6.0
	*L_p_* = 4	4.0	5.3	6.3	7.3	8.1
	*L_p_* ≈ *N*/2	5	7.3	9.7	12	14.5
	*L_p_* ≈ *N*	5.3	8	10.5	13.1	13.8
*R_h_* = (5/3*R_g_*)^1/2^	1, 0.794	4.2	5.0	5.6	6.1	6.6
	12.5, 0.720	3.8	4.4	4.9	5.3	5.7
	25, 0.700	3.6	4.2	4.6	5.0	5.3
	125, 0.653	3.4	3.9	4.3	4.6	4.9
	225, 0.636	3.3	3.7	4.1	4.4	4.6
	525, 0.607	3.2	3.6	4.0	4.3	4.5
	1000, 0.592	3.1	3.5	3.8	4.1	4.3
	10,000, 0.524	2.8	3.1	3.4	3.6	3.8
*R_e-e_* = 3.1*R_h_*	1, 0.794	13.1	15.4	17.3	18.9	20.3
	12.5, 0.720	11.8	13.7	15.2	16.5	16.6
	25, 0.700	11.3	13.0	14.3	15.5	16.5
	125, 0.653	10.6	12.1	13.3	14.3	15.2
	225, 0.636	10.2	11.6	12.7	13.6	14.4
	525, 0.607	10.0	11.3	12.3	13.2	14.0
	1000, 0.592	9.7	11.0	11.9	12.7	13.4
	10,000, 0.524	8.8	9.8	10.5	11.2	11.7

## Data Availability

This study does not report any new data, only results of the indicated calculations.
